# The treatment of diseases related to balance disorders in the elderly and the effectiveness of vestibular rehabilitation

**DOI:** 10.1016/S1808-8694(15)30071-9

**Published:** 2015-10-19

**Authors:** Roseli Saraiva Moreira Bittar, Lucinda Simoceli, Maria Elisabete Bovino Pedalini, Marco Aurélio Bottino

**Affiliations:** a Doctor in Medicine, Assistant doctor in the Otoneurology unit at the HCFMUSP; b Graduate doctoral student in the Otorhinolaryngology Discipline at FMUSP. Otorhinolaryngologist in the postgraduate course on Sciences at FMUSP; c Doctor on Sciences, speech therapist in charge of the Vestibular Rehabilitation outpatient unit at the HCFMUSP; d Doctor in Medicine, Assistant doctor in the Otoneurology unit at the HCFMUSP. Otorhinolaryngology Department - Clinical Hospital of the Sao Paulo University Medical School

**Keywords:** balance diseases, elderly, vestibular rehabilitation

## Abstract

The aim of this study was to assess the impact of adequate treatment of concomitant diseases in the elderly undergoing Vestibular Rehabilitation (VR). **Method:** 52 elderly patients with complaints of vertigo and/or imbalance requiring VR participated in this prospective study. The trial was designed as an open clinical assay at the Ear Nose and Throat Department Geriatric Otoneurology Clinic, and was done between 2003 and 2005. Patients were compared with the total group of elderly individuals treated with VR during the same period. **Results:** 65 diseases were diagnosed in the study group, an average 1.25 diseases per patient. After the treatment of these diseases, patients underwent VR. The effectiveness of VR (remission and partial improvement rates) was 84.5% in the study group against 81.8% in the control group, which was not significant. Remission of symptoms, however, was present in 69.2% of the study group against 43.18% of the control group, which was statistically significant. **Conclusion:** The difference in the effectiveness of VR in both groups highlights the importance of the etiological treatment of concomitant diseases in patients with vestibular disorders.

## INTRODUCTION

The increased number of elderly people in the world has significantly changed the strategy against diseases in this age group. Currently there are people that reach 80 to 90 years in good medical conditions, although “natural senescence” is still unavoidable. The balance system is affected, there is loss of neurons and vestibular hair cells, limitations of joints, reduced visual acuity, and cognition difficulties.^1^, ^2^ Some authors consider balance disorders as part of a geriatric syndrome, given their frequency.^3^ Vestibular rehabilitation therapy (VRT) has filled a gap in the treatment of these patients with mobility limitations, reducing the rate of falls in the elderly, and improving spatial orientation and well-being.

The available treatment today controls most of the diseases such as diabetes, lipid metabolic dysfunction, thyroid diseases, and heart conditions. Lack of balance secondary to or concomitantly with those diseases usually brings these patients to the otolaryngologist’s office, who can offer support through medical drugs and VRT. Frequently improvements fall short of the desired aims due to the diseases that are associated with unbalance, and to the use of a variety of symptomatic drugs that may cause undesirable adverse effects.^4^, ^5^

In a previous paper we reported a 71.43% effectiveness rate of VRT in elderly patients seen at our unit.^6^ A similar rate is seen in other age groups, showing that elderly patients have a similar response to treatment as the general otoneurological population.^7^ To attain even better results, we organized a special outpatient unit for persons aged over 65 years within our outpatient clinic, where patients received a focused geriatric assessment and care. Not only does the patient benefit from the full diagnostic apparatus available at our otoneurology outpatient unit, but also receives clinical treatment geared to the specificities of his or her age. We expected that correction of comorbidities by using medical drugs, diet or therapy would improve the response rate to the specific treatment of vestibular diseases.^8^

## OBJETIVE

Our aim was to assess the impact on the effectiveness of VRT of correcting various comorbidities present in the geriatric group of patients.

## METHOD

Subjects aged over 65 years, seen at the geriatric otoneurology outpatient unit between 2003 and 2005, were included in this study. All of the work abided by the current ethical guidelines of the institution. The study was an open clinical trial, which was approved by the Research Ethics Committee for the analysis of research projects of the CAPPESQ, under protocol number 1027/03.

A clinical history was taken and a complete otoneurological exam was made of all patients. The exam includes complete audiometry, a report of spontaneous nystagmus, ocular movement, positional and caloric tests. Standard laboratory tests included a complete blood count, fasting blood sugar, measurement of thyroid hormones, the lipid profile, blood zinc, and syphilis serology. The glucose tolerance test was done in suspected cases and included three-hour blood insulin.^9^

After defining the otoneurological condition, patients that required VRT were first investigated for systemic diseases. A cardiologic evaluation was done in the event of vascular system disorders to correct conditions such as arrhythmia, carotid sinus sensitivity, or orthostatic intolerance.10 Patients with positive laboratory tests were assessed and referred to the geriatric clinic for management with diet and/or medication.

Only after adequate treatment of the abovementioned conditions were the patients referred to VRT, which was adapted to the individual needs of each elderly patient. Treatment was given using the Cawthorne-Cooksey basic protocols, work with the vestibulo-ocular reflex (VOR) and Norré exercises.^11^ Patients were informed about the causes of their balance difficulties, about basic vestibular physiology and incorrect habits that interfere with vestibular compensation phenomena. We chose to apply the exercises at the patients’s households once or twice a day; patients were asked to visit the clinic once every fifteen days or monthly according to their need, totaling 4 to 5 visits. Total treatment time was 3 months.

Qualitative evaluation of the response to treatment was done using a visual analog scale. Remission was considered as resolution of symptoms, improvement was that between 50 and 90%, and no improvement was that below 50%.6 Results were compared to those obtained from the full sample of patients treated by VRT at the outpatient clinic during the same time period using the chi-squared test at p<0.05.

## RESULTS

The study included 52 elderly subjects aged between 65 and 95 years (mean age - 74 years; standard deviation - 6.91) that completed VRT.

Table 1 shows the clinical profile of the patients and the comorbidities. We diagnosed 65 diseases associated with unbalance in 52 subjects, a mean 1.25 occurrences per elderly patient. The most frequent comorbidities were those associated with metabolic disorders that together add up to 50.6% of patients. These conditions include: dyslipidemias (29.2%), sugar metabolism (13.8%), and thyroid conditions (7.6%). Vascular diseases were found in 32.3% of patients, and included vertebrobasilar insufficiency (VBI), orthostatic intolerance, and cardiac arrhythmias. Neurological and psychiatric conditions affected 16.9% of patients.

**Table 1:** Comorbidities in elderly patients referred for VRT. Some patients had more than one associated disease. * VBI = vertebrobasilar insufficiency; OI = orthostatic intolerance

UNBALANCE-ASSOCIATED COMORBIDITIES ^n^	
Vascular (IVB*, IOT*. arrhythmias)	21 (32,3%)
Dyslipimias	19 (29,2%)
Sugar metabolism disorders	9 (13,8%)
Psychiatric	8 (12,3%)
Thyroidal disorders	5 (7,6%)
Neurologic	3(4,6%)

**Figure 1:**
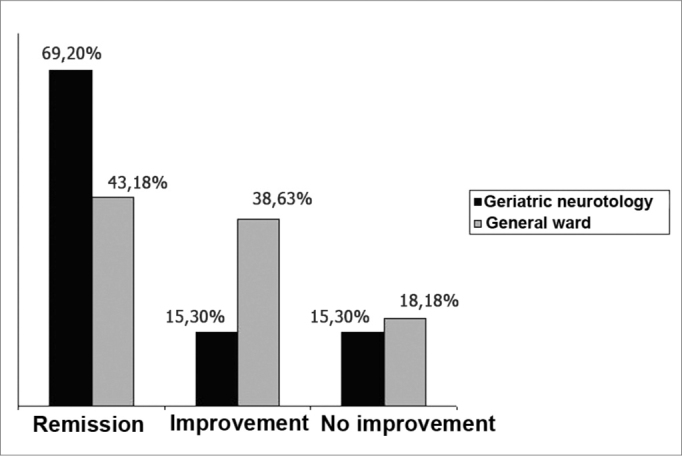
Effectiveness of response to VRT in elderly patients from the outpatient clinic and the geriatric otoneurology unit.

Table 2 shows the distribution of elderly patients that underwent VRT at the outpatient clinic and the geriatric otoneurology unit. Figure 1 shows the effectiveness of VRT (sum of remission and partial improvement rates), which was 84.5% in the geriatric otoneurology group against 81.8% in the outpatient clinic; this difference was not significant. In the geriatric otoneurology group we found remission of symptoms in 69.2% of patients against 43.18% remission in the outpatient clinic group (p<0.05). The symptom remission rate was 26.02% higher in the sample group.

**Table 2:** Distribution of the total number of elderly patients in the geriatric otoneurology unit and the outpatient clinic that concluded the VRT protocol.

	REMISSION 100%	IMPROVEMENT Between 50 and 90%	NO IMPROVEMENT <50%	TOTAL
Geriatric Neurotology	36	8	8	52 (54,1%)
Elderly in the General Ward	19	17	8	44 (45,8%)

Total of patients	55 (57,2%)	25 (26,04%)	16 (16,66%)	96 (100%)

Analysis of results using the chi-squared test revealed that improvement of patients from the geriatric otoneurology unit was significantly superior compared to the total group of patients: x^2^= 7.86; (p<0.02%).

## DISCUSSION

VRT is considered the best treatment for balance disorders in the elderly.^6^ This statement is valid as long as we consider that it is pointless to treat the symptoms of balance disorders when other diseases typical of this age group are not treated. Metabolic and vascular diseases are common in these patients, and maintain poor cochleovestibular homeostasis and the resulting balance disorders. Elderly patients improve just as well as younger patients when adequate treatment is given.^6^, ^12^, ^13^ Our view of the elderly as “whole” persons means that body unbalance requires multidisciplinary treatment.

As previously reported, we noted that elderly patients with balance disorders usually have more than one disease that may cause dizziness. We found 1.25 comorbidities per rehabilitated patient, which confirms our previously published data.^5^ We highlight the importance of seeking not only a specific diagnosis but all of the clinical variables that may be altered. Associated diseases negatively affect the performance of the vestibular system, at least in part generating clinical symptoms and interfering with central compensation. Frequently antivertigo drugs interfere still further with homeostasis and vestibular adaptation; symptoms not only do not improve but may even worsen. VRT is individualized, and offers best results when associated with treatment of all the concomitant clinical variables. This is corroborated by the significant improvement in VRT responses that reaching significant remission rates after correcting concomitant diseases. Improvements in the visual analog scale correlate directly with adaptation of elderly patients to daily activities and their environment, respecting limits imposed by natural senescence but without restricting integration into family and social life.

Some authors believe that elderly patients require extra treatment time compared to younger patients, and that full vestibular compensation is never attained. Other papers have concluded that age is not a significant factor, and does not change the response rate to VRT.^6^, ^11^, ^12^ Our results suggest that elderly patients not only respond favorably to VRT but also respond more effectively to treatment when we first treat underlying diseases.8 The moment at which VRT is recommended is crucial for success. Unsatisfactory responses may be due to VRT being applied at the wrong moment, when the patient has not yet reached a favorable clinical state.

Our series shows that vascular diseases were not the main villains causing unbalance in elderly patients. Metabolic diseases, particularly dyslipidemias, respond for over 50% of comorbidities that affect balance in this age group. Altered sugar metabolism, which is so frequently found in patients presenting dizziness,8 is common in the elderly. Some patients had reactive hyperinsulinemia and hypoglycemia, but diabetes mellitus effectively predominates. We underline that early management of metabolic dysfunction not only improves the response to VRT but also prevents neuropathies and vascular diseases.

## CONCLUSION

The effectiveness of VRT, where symptomatic remission rates improved by 26.02%, highlights the importance of the etiological treatment of coexisting diseases in patients with vestibular diseases, confirming our hypothesis that etiological treatment allied to VRT is the preferred management for these patients.
